# Monocyte‐to‐Albumin Ratio Predicts the Functional Outcome of Adults With Status Epilepticus: An Observational Study

**DOI:** 10.1002/brb3.71204

**Published:** 2026-01-15

**Authors:** Jie Fu, Lilei Peng, Jinglun Li, Jun Liu, Shan Zeng

**Affiliations:** ^1^ Department of Neurology the Affiliated Hospital of Southwest Medical University Luzhou Sichuan China; ^2^ Department of Neurology the Wangcang County People's Hospital Guangyuan Sichuan China; ^3^ Department of Neurosurgery the Affiliated Hospital of Southwest Medical University Luzhou Sichuan China

## Abstract

**Background:**

Status epilepticus (SE) is a serious neuro‐emergency that is often associated with unfavorable outcomes. It is known that inflammation is involved in the pathogenesis of SE, and various inflammatory markers have been suggested to be related to SE prognosis. Monocyte‐to‐albumin ratio (MAR), a novel biomarker of systemic inflammation, is derived from monocyte count and albumin levels. In this study, we sought to explore whether MAR could serve as a predictor of functional outcomes in SE.

**Methods:**

This retrospective study collected the data of adult patients with SE. Functional outcomes of SE patients were evaluated by using the Modified Rankin Scale (mRS). Multivariable logistic regression analysis was performed to investigate the association of MAR with SE outcomes. Moreover, receiver operating characteristic (ROC) curve analysis was carried out to determine the optimal MAR threshold for predicting poor SE outcomes.

**Results:**

This study included 163 SE patients. Poor outcome at discharge was observed in 39.3% (64/163). Multivariate analysis showed that higher MAR at admission was independently related to unfavorable outcomes of SE patients (odds ratio: 1.092; 95% confidence interval, 1.023–1.166; *p* = 0.008). ROC curve analysis demonstrated that MAR could predict poor SE outcomes, with an area under the curve of 0.717 (95% CI: 0.638–0.795, *p* < 0.001). The optimal predictive cutoff point of MAR for poor SE outcomes was 13.78 (sensitivity 59.38%, specificity 73.74%).

**Conclusion:**

Higher MAR at admission is closely correlated with an elevated risk of poor functional outcomes at discharge of SE patients. Our data suggest that MAR may be a promising and easily measurable marker for predicting short‐term SE outcomes.

## Graphical abstract

Our study aimed to investigate whether the monocyte‐to‐albumin ratio (MAR), a novel marker of systemic inflammation, could serve as a predictor of functional outcomes in status epilepticus (SE).

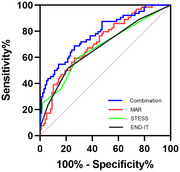

## 1 Introduction

Status epilepticus (SE) is a serious neuro‐emergency, which is defined as a disorder resulting from the failure of seizure termination or from triggering relative mechanisms that cause extended seizures (Kapur 2025). The annual incidence of SE ranges from 10 to 40 cases per 100,000 people (Hill et al. 2017). SE carries a high mortality risk and often leads to lasting physical and cognitive deficits, placing a heavy burden on patients and those who care for them (King‐Stephens et al. 2020). Developing useful indicators for predicting SE outcomes is of great importance, which contributes to recognizing SE patients with high risks of unfavorable outcomes and thus timely adjusts and optimizes clinical decision‐making.

Previous studies have reported some score systems to predict SE prognosis, such as the Status Epilepticus Severity Score (STESS) and the Encephalitis‐NCSE‐Diazepam resistance‐Image abnormalities‐Tracheal intubation (END‐IT). The STESS scale includes four factors: prior history of seizures, age, seizure type, and consciousness status, which is used for early mortality risk stratification in SE (Rossetti et al. 2006). The END‐IT score system consists of five parameters: encephalitis, non‐convulsive SE, resistance to diazepam, tracheal intubation, and brain lesions, which is utilized to assess functional outcome recovery of SE patients (Yuan et al. 2018). However, the applicability of these scales is restricted under specific conditions. For instance, the evaluation of the STESS scale relies on the previous seizure history of patients, which may be unavailable if patients have impaired consciousness and their relatives are absent. In addition, there is controversy over the predictive ability of the END‐IT scale for functional outcomes in SE patients. A study from Gao et al. (2016) indicated that the END‐IT score outperformed both STESS and the epidemiology‐based mortality score in status epilepticus (EMSE) in terms of discriminative power and predictive accuracy for the assessment of SE outcomes, while other investigators argued that the END‐IT scale exhibited poor performance in predicting SE prognosis (Lin et al. 2019). Hence, it is necessary to develop easily available and reliable markers to enhance existing scoring systems for predicting SE outcomes.

In recent years, a neuroinflammation has attracted increasing attention and is suggested to be involved in the pathophysiological processes of SE. SE could trigger a neuroinflammatory cascade in the brain, and neuroinflammatory response may facilitate recurrent seizures (Wang and Chen 2018). Prior investigations have revealed the close association of inflammatory biomarkers with SE prognosis (Liao et al. 2023; Sutter et al. 2015). The monocyte‐to‐albumin ratio (MAR), a new marker of systemic inflammation, has been indicated to be associated with multiple diseases, such as cardiovascular diseases (Zhang et al. 2021) and tumors (Zhao et al. 2023). Of note, our previous study found that MAR could be an independent predictor for hematoma expansion in patients with spontaneous intracerebral hemorrhage (Fu et al. 2024). However, the correlation between MAR and SE outcomes has not been uncovered. Thus, our present study sought to explore whether MAR could predict functional outcomes in SE.

## 2 Materials and Methods

### 2.1 Study Population

We carried out a retrospective analysis on SE patients admitted to the affiliated hospital of Southwest Medical University from January 2020 to December 2024 and to the Wangcang County People's Hospital from January 2023 to December 2024. The ethics committee of the affiliated hospital of Southwest Medical University approved the present study (Approval No. KY2024477), and this study was performed in agreement with the Helsinki Declaration principles. Written consent was acquired from the individual patient or his/her family members. This investigation enrolled patients aged over 18 years old who were diagnosed with SE. SE was defined as either a seizure lasting longer than 5 min or recurrent seizures without full recovery of consciousness between episodes (Lin et al. 2019). We ruled out patients whose SE resulted from post‐cardiac arrest hypoxic‐ischemic encephalopathy and those lacking complete clinical data.

### 2.2 Data Collection

To investigate the potential factors impacting clinical outcomes at discharge, clinical information and laboratory examination results were collected. Clinical information included age, sex, prior history of seizure, SE etiology, presence of fever at onset, status epilepticus severity score (STESS) at SE onset, Encephalitis‐NCSE‐Diazepam resistance‐Image abnormalities‐Tracheal intubation (END‐IT) score, and comorbidities (hypertension, diabetes mellitus, and previous stroke). The STESS scale assesses SE severity based on four factors: age, history of seizures, type of seizure, and consciousness status (Rossetti et al. 2006). The END‐IT score system consists of five parameters: encephalitis, non‐convulsive SE, resistance to diazepam, tracheal intubation, and brain lesions (Yuan et al. 2018). Data of laboratory examination were collected within 24 h of admission, including red blood cell count, hemoglobin, white blood cell count, neutrophil count, lymphocyte count, monocyte count, platelet count, and albumin. The neutrophil‐to‐lymphocyte ratio (NLR), monocyte‐to‐lymphocyte ratio (MLR), platelet‐to‐lymphocyte ratio (PLR), and neutrophil‐to‐albumin ratio (NAR) were defined as the ratio of neutrophil count to lymphocyte count, the ratio of monocyte count to lymphocyte count, the ratio of platelet count to lymphocyte count, and the ratio of neutrophil count to albumin level, respectively. Monocyte‐to‐albumin ratio (MAR) was computed as (monocyte count × 1000) divided by the albumin level (Fu et al. 2024).

### 2.3 Outcomes

The Modified Rankin Scale (mRS) scores were used to evaluate the functional outcomes at discharge from the hospital. A poor outcome was defined as an mRS score of 3 or higher (including death), whereas a good outcome corresponded to an mRS score below 3 (Qiao et al. 2022).

### 2.4 Statistical Analysis

The Shapiro–Wilk test was employed to evaluate whether continuous variables followed a normal distribution. Continuous variables of normal distribution were analyzed with the t‐test, while continuous variables of skew distribution were compared with the Mann–Whitney *U*‐test. Categorical variables were analyzed with the chi‐square (*χ*2) test. Variables with *p* < 0.05 in the univariate analysis entered into the multivariate logistic regression model. We used the variance inflation factors (VIF) to evaluate multicollinearity. The variables with tolerance > 0.1 and VIF < 5 were selected for further multivariate analysis. Receiver operating characteristic (ROC) curve analysis was utilized to investigate predictive value, and the DeLong test was applied to compare the areas under ROC curves. The optimal MAR cutoff value was identified using the Youden index. The propensity score matching process was conducted by using a logistic regression model with exposure assignment (based on whether MAR was above or below the cutoff value) as the dependent variable and potential confounders as independent variables. Statistical analysis was performed using GraphPad Prism 9.0 software, SPSS 26.0 software and MedCalc 22.0 software, and *p* < 0.05 demonstrated statistical significance.

## 3 Results

### 3.1 Clinical Characteristics of Patients With SE

A total of 163 SE patients were ultimately included in our study. Patients were divided into two groups on the basis of functional outcomes at discharge. Clinical information and laboratory data of all patients were demonstrated and compared in Table 1. Among 163 SE patients in this study, 64 (39.3%) experienced poor outcomes at discharge. The MAR levels of SE patients with poor outcomes were significantly higher than those of SE patients with good outcomes (*p* < 0.001). In addition, compared to SE patients with good outcomes, patients with poor outcomes were more likely to have older age, higher STESS at SE onset and END‐IT score, increased levels of white blood cell count, neutrophil count, monocyte count, NLR, MLR, and NAR, as well as lower levels of platelet count and albumin (all *p* < 0.05). No obvious differences were found in male ratio, SE etiology, presence of fever at onset, seizure history, ratios of patients with hypertension, diabetes mellitus, or prior history of stroke, red blood cell count, hemoglobin, lymphocyte count, and PLR between SE patients with favorable outcomes and those with unfavorable outcomes (all *p* > 0.05).

**TABLE 1:** Clinical characteristics and laboratory data between SE patients with good outcomes and poor outcomes at discharge.

Variable	Good‐outcome (*n* = 99)	Poor‐outcome (*n* = 64)	*p*
Male (*n*, %)	59 (59.6)	36 (56.3)	0.672
Age, years, median (IQR)	50.0 (28.5–65.0)	60.5 (42.0–71.0)	0.011
SE etiology (*n*, %)			0.532
Cryptogenic	42 (42.4)	24 (37.5)	
Symptomatic	57 (57.6)	40 (62.5)	
Stroke etiology of SE (*n*, %)	10 (10.1)	10 (15.6)	0.294
Infectious etiology of SE (*n*, %)	14 (14.1)	12 (18.8)	0.433
Fever at onset (*n*, %)	26 (26.3)	23 (35.9)	0.188
No history of seizures (*n*, %)	58 (58.6)	38 (59.4)	0.920
STESS at SE onset, median (IQR)	2.0 (1.0–3.0)	3.0 (2.0–4.3)	< 0.001
END‐IT score, median (IQR)	1.0 (1.0–1.0)	2.0 (1.0–2.0)	< 0.001
Comorbidities (*n*, %)			
Hypertension	34 (34.3)	30 (46.9)	0.110
Diabetes mellitus	11 (11.1)	12 (18.8)	0.171
Previous stroke	15 (15.2)	10 (15.6)	0.935
Red blood cell count (×10^6^ /µL), median (IQR)	4.41 (4.08–4.98)	4.36 (3.79–4.79)	0.070
Hemoglobin (g/L), median (IQR)	134 (122–153)	130 (121–143)	0.139
White blood cell count (×10^3^ /µL), median (IQR)	9.00 (6.78–11.55)	11.05 (6.96–15.33)	0.014
Neutrophil count (×10^3^ /µL), median (IQR)	6.94 (4.18–9.47)	9.12 (5.41–11.67)	0.014
Lymphocyte count (×10^3^ /µL), median (IQR)	1.33 (0.96–1.94)	1.08 (0.83–1.51)	0.079
Monocyte count (×10^3^ /µL), median (IQR)	0.43 (0.33–0.59)	0.64 (0.42–0.89)	< 0.001
Platelet count (×10^3^ /µL), median (IQR)	209 (172–268)	185 (148–246)	0.025
Albumin (g/L), mean (SD)	42.7 (5.2)	39.5 (5.9)	< 0.001
NLR, median (IQR)	4.10 (2.39–8.91)	6.94 (3.50–10.63)	0.024
MLR, median (IQR)	0.33 (0.20–0.51)	0.46 (0.33–0.96)	0.001
PLR, median (IQR)	159.48 (104.96–243.90)	143.15 (93.80–260.10)	0.755
NAR, median (IQR)	0.17 (0.10–0.22)	0.22 (0.14–0.31)	0.001
MAR, median (IQR)	9.92 (7.58–14.24)	15.66 (10.39–20.91)	< 0.001

**Abbreviations**: END‐IT, encephalitis‐ncse‐diazepam resistance‐image abnormalities‐tracheal intubation; IQR, interquartile range; MAR, monocyte‐to‐albumin ratio; MLR, monocyte‐to‐lymphocyte ratio; NAR, neutrophil‐to‐albumin ratio; NLR, neutrophil‐to‐lymphocyte ratio; PLR, platelet‐to‐lymphocyte ratio; SD, standard deviation; SE, status epilepticus; STESS, status epilepticus severity score.

### 3.2 Multivariate Logistic Regression Analysis

We conducted a multivariate logistic regression analysis to explore possible risk factors associated with functional outcomes of SE patients, which was presented in Table 2. In detail, univariable analysis identified age, STESS, END‐IT, white blood cell count, neutrophil count, monocyte count, platelet count, albumin, NLR, MLR, NAR, and MAR as prognostic factors for poor outcomes of SE patients (all *p* < 0.05). After multicollinearity assessment, age, STESS, END‐IT, platelet count, white blood cell count, NLR, NAR, and MAR were included in further multivariable analysis, which indicated that MAR was independently associated with poor SE outcomes (odds ratio: 1.092; 95% confidence interval, 1.023–1.166; *p* = 0.008). In addition, STESS, END‐IT, and platelet count remained significant in the multivariate analysis (*p* = 0.047, *p* = 0.010, and *p* = 0.009, respectively).

**TABLE 2:** Multivariate analysis of predictors for poor functional outcomes in SE patients.

Variable	OR	95% CI	*p*
Age	1.004	0.979–1.030	0.745
STESS at SE onset	1.476	1.005–2.168	0.047
MAR	1.092	1.023–1.166	0.008
END‐IT score	1.762	1.142–2.718	0.010
White blood cell count	1.053	0.897–1.235	0.529
NLR	1.032	0.965–1.105	0.357
NAR	0.339	0.000–877.387	0.787
Platelet count	0.992	0.987–0.998	0.009

**Abbreviations**: CI, confidence interval; END‐IT, encephalitis‐NCSE‐diazepam resistance‐image abnormalities‐tracheal intubation; MAR, monocyte‐to‐albumin ratio; NAR, neutrophil‐to‐albumin ratio; NLR, neutrophil‐to‐lymphocyte ratio; OR, odds ratio; SE, status epilepticus; STESS, status epilepticus severity score.

### 3.3 The Predictive Power of MAR for Functional Outcomes at Discharge in SE Patients

**FIGURE 1:**
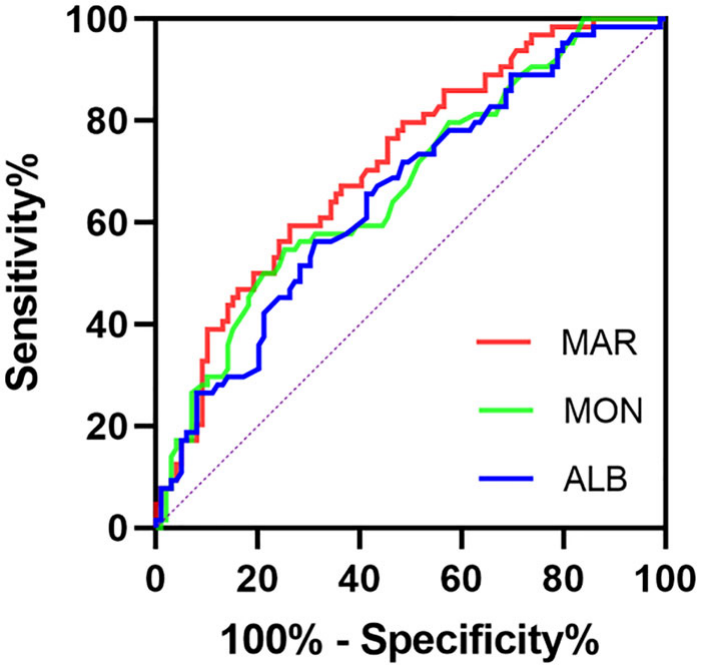
Receiver operating characteristic curve of MAR, MON and ALB to predict poor functional outcomes at discharge in SE patients. **Abbreviations**: ALB, albumin; MAR, monocyte‐to‐albumin ratio; MON, monocyte count.

The ROC curve was conducted to assess the predictive ability of MAR for poor outcomes at discharge in SE patients. The area under the ROC curve of MAR was 0.717 (95% confidence interval: 0.638–0.795, *p* < 0.001) for poor outcomes, which was greater than those of monocyte count (0.673, 95% confidence interval: 0.589–0.757, *p* < 0.001) and albumin (0.656, 95% confidence interval: 0.571–0.741, *p* = 0.001) (Figure 1). The difference between areas MAR vs. monocyte count reached statistical significance (0.044, 95% confidence interval: 0.015–0.073, *p* = 0.003), which was not observed in the difference between areas MAR vs. albumin (0.061, 95% confidence interval: −0.044 to 0.166, *p* = 0.255). The optimal predictive cutoff point for poor outcomes at discharge of SE patients by MAR was 13.78 (sensitivity 59.38%, specificity 73.74%). Subsequently, 163 patients with SE were classified into two groups according to the MAR cutoff value (MAR < 13.78 and MAR ≥ 13.78). We compared the data of poor outcomes at discharge across both groups. As anticipated, a higher proportion of poor outcomes at discharge was observed in SE patients with MAR ≥ 13.78 (*p* < 0.001) (Table 3). Given the differences in clinical characteristics between the two groups, a propensity score matching analysis was used to correct for potential imbalance effects. After matching, the two groups were adequately balanced, and SE patients with MAR ≥ 13.78 still had a remarkably higher ratio of poor outcomes at discharge than those with MAR < 13.78 (*p* < 0.001) (Supplementary Table S1).

**TABLE 3:** Clinical characteristics and poor functional outcomes between patients with different levels of MAR.

	MAR		*P*
	< 13.78 (*n* = 99) ≥ 13.78 (*n* = 64)		
Male (*n*, %)	58 (58.6)	37 (57.8)	0.922
Age, years, median (IQR)	51.0 (31.0–66.0)	58.5 (30.8–70.3)	0.194
SE etiology (*n*, %) 0.978			
Cryptogenic	40 (40.4)	26 (40.6)	
Symptomatic	59 (59.6)	38 (59.4)	
Stroke etiology of SE (*n*, %)	11 (11.1)	9 (14.1)	0.575
Infectious etiology of SE (*n*, %)	18 (18.2)	8 (12.5)	0.333
Fever at onset (*n*, %)	13 (13.1)	36 (56.3)	< 0.001
No history of seizures (*n*, %)	56 (56.6)	40 (62.5)	0.452
STESS at SE onset, median (IQR)	2.0 (1.0–3.0)	2.5 (2.0–4.0)	0.026
END‐IT score, median (IQR)	1.0 (1.0–2.0)	1.0 (1.0–2.0)	0.263
Comorbidities (*n*, %)			
Hypertension	38 (38.4)	26 (40.6)	0.775
Diabetes mellitus	11 (11.1)	12 (18.8)	0.171
Previous stroke	18 (18.2)	7 (10.9)	0.210
Poor outcomes at discharge (*n*, %)	26 (26.3)	38 (59.4)	< 0.001

**Abbreviations**: END‐IT, encephalitis‐NCSE‐diazepam resistance‐image abnormalities‐tracheal intubation; IQR, interquartile range; MAR, monocyte‐to‐albumin ratio; SE, status epilepticus; STESS, status epilepticus severity score.

### 3.4 Comparison Among Baseline MAR, STESS Score and END‐IT Score as Predictors for Poor Outcomes at Discharge in SE Patients

**FIGURE 2:**
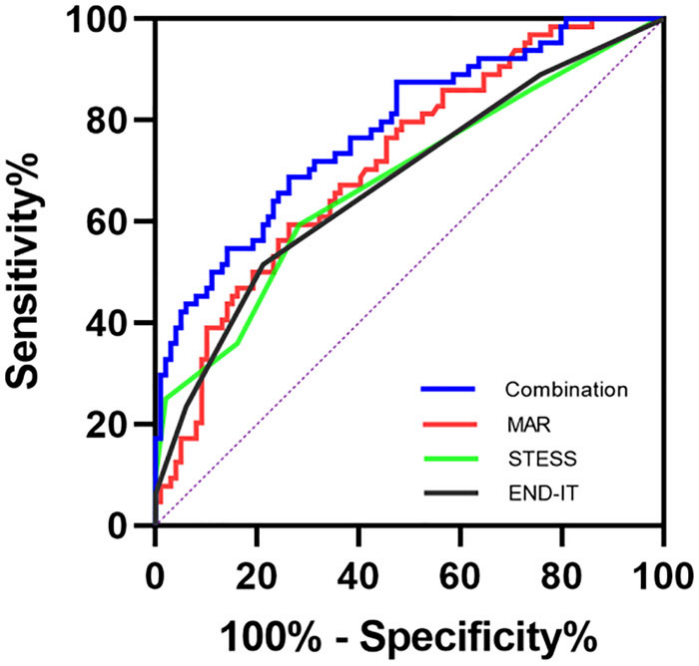
Receiver operating characteristic curve of MAR, STESS score, END‐IT score and the combination of MAR, STESS score, and END‐IT score to predict poor functional outcomes at discharge in SE patients. **Abbreviations**: END‐IT, encephalitis‐NCSE‐diazepam resistance‐image abnormalities‐tracheal intubation; MAR, monocyte‐to‐albumin ratio; STESS, status epilepticus severity score.

We further compared the predictive power of STESS, END‐IT, and MAR for poor outcomes at discharge in SE patients. The area under the ROC curve of MAR was 0.717 (95% confidence interval: 0.638–0.795, *p* < 0.001) for poor outcomes, which was slightly larger than those of STESS (0.680, 95% confidence interval: 0.593–0.766, *p* < 0.001) and END‐IT (0.679, 95% confidence interval: 0.593–0.764, *p* < 0.001), but the differences did not reach statistical significance (difference between areas MAR vs. STESS: 0.037, 95% confidence interval: −0.076 to 0.150, *p* = 0.519; difference between areas MAR vs. END‐IT: 0.038, 95% confidence interval: −0.073 to 0.149, *p* = 0.500). Additionally, we also investigated the predictive ability of the combination of MAR, STESS, and END‐IT for poor outcomes at discharge in SE patients. The area under the ROC curve of the combination of MAR, STESS, and END‐IT was 0.781 (95% confidence interval: 0.709–0.853, *p* < 0.001) for poor outcomes, which was larger than those of MAR, STESS, and END‐IT alone (Figure 2). Collectively, our results suggested that the predictive ability of MAR at admission for poor outcomes at discharge in SE patients was not inferior to that of the STESS score or the END‐IT score. Moreover, combining MAR, STESS, and END‐IT improved the identification of SE patients with poor outcomes.

## 4 Discussion

In the present study, we investigated the predictive capacity of MAR for short‐term functional outcomes in SE, which suggested that higher MAR at admission was an independent predictor for unfavorable outcomes at discharge in SE. Notably, this was the first research to explore the association of MAR with SE outcomes.

Inflammation plays a key role in the pathophysiology of SE. SE could damage the blood‐brain barrier (BBB) and trigger the activation of glial cells, causing the release of pro‐inflammatory mediators, further enhancing neuronal excitability and lowering the seizure threshold, leading to recurrent seizures and perpetuating a vicious cycle (Rejdak et al. 2023). Multiple inflammatory biomarkers have been evidenced to hold predictive value for functional outcomes in SE patients. Albumin is one of the acute‐phase proteins, which has been indicated to exert anti‐inflammatory and antioxidant effects (Deng et al. 2021; Wiedermann 2007). Under inflammation, albumin concentrations drop due to both diminished liver synthesis and increased capillary leakage (Zhang et al. 2021). Several studies have unveiled the potential relationship between albumin levels and SE prognosis. Liao et al. (2023) analyzed the clinical information of 51 patients with SE and found that admission albumin level was an independent predictive factor for SE outcomes at discharge. Moreover, a study by Misirocchi et al. (2025) indicated that hypoalbuminemia was closely associated with in‐hospital and 6‐month mortality in SE patients. Monocytes are key components of the innate immunity and exert a vital role in modulating inflammatory responses (Austermann et al. 2022). It is reported that infiltrating monocytes after SE onset could promote inflammatory reaction in the central nervous system, and decreasing monocyte infiltration may improve seizure‐associated outcomes (Bosco, Tian, and Wu 2020). As mentioned above, it is rational to believe that the MAR, derived from monocyte count and albumin levels, may represent a potential and reliable indicator for predicting functional outcomes in SE patients. Nevertheless, the predictive capacity of MAR for SE outcomes remains unexplored in current studies, highlighting a critical research gap that warrants investigation. Thus, our study bridged this gap, which indicated that an increased MAR level at admission was an effective predictor for unfavorable short‐term functional outcomes in SE patients.

Of note, some score systems have been developed to predict SE prognosis, such as the STESS score and the END‐IT score. The STESS scale, proposed by Rossetti et al. (2006), has been widely applied to grade SE severity and predict SE outcomes. The END‐IT score is utilized to assess functional outcome recovery of SE patients (Yuan et al. 2018). However, current evidence presents conflicting views on the predictive validity of these scales for functional outcomes of SE patients. Shen et al. (2024) reported that the STESS scale could be useful for predicting in‐hospital death of SE patients but was poorly associated with functional outcomes at discharge. On the contrary, the study by Krejzar et al. (2024) conducted a single‐center prospective pilot study including 30 SE patients, which revealed the good predictive ability of the admission STESS scale regarding SE outcomes at hospital discharge. Additionally, the study by Gao et al. (2016) indicated that the END‐IT score outperformed both STESS and EMSE regarding discriminative power and predictive accuracy for evaluating SE outcomes, while other research argued that the END‐IT scale exhibited poor ability for predicting SE prognosis (Lin et al. 2019). In our study, we observed that both the STESS scale and the END‐IT scale were effective predictors for SE outcomes at discharge, but our results warrant additional studies, given the limited sample size of our research. As we know, the STESS score consists of four components, including age, history of seizures, seizure type, and level of consciousness, which can be obtained based on data from medical records. Nevertheless, under some specific conditions, its application may be limited. For instance, SE patients who suffer from consciousness impairment could not offer their previous seizure history, especially when their family members or caregivers are absent, which causes the inability to acquire the parameter of past seizure history, further leading to the unavailability of the STESS scale. Fortunately, our study uncovered the predictive validity of MAR, a simple and easily available hematological biomarker, for SE outcomes. Furthermore, we also noted that the combination of MAR, STESS, and END‐IT exhibited greater predictive power for unfavorable SE outcomes compared to either marker alone. Therefore, MAR may effectively supplement the present scoring systems.

In this study, we also included some other established inflammatory biomarkers, such as NLR, MLR, PLR, and NAR. These biomarkers have been indicated to be potential factors for predicting the outcomes of multiple neurological disorders, including status epilepticus (Olivo et al. 2023), ischemic stroke (Guo et al. 2024), and traumatic brain injury (Li et al. 2024), etc. However, our study did not suggest the predictive value of these markers in SE outcomes, while MAR was an effective predictor for SE outcomes at hospital discharge that outperformed NLR, PLR, MLR, and NAR in predictive power. Interestingly, a very recent study reported MAR's superior predictive capacity for cardiovascular diseases over multiple inflammatory biomarkers (Jin et al. 2025). The possible reason for MAR's superior predictive value may be that MAR integrates both monocyte‐mediated pro‐inflammatory activity and albumin‐mediated nutritional and anti‐inflammatory defense mechanisms and thus represents a more comprehensive and stable biological indicator.

Some limitations were noted in the current study. First, this retrospective study was performed on a limited sample. Second, we only tested baseline MAR, and dynamic MAR monitoring is lacking. Third, this study focused on short‐term SE outcomes at discharge, and future investigations with extended follow‐up periods are needed to assess the predictive value of MAR for long‐term prognosis.

## 5 Conclusion

Our study indicated that MAR at admission was an effective predictor for SE outcomes at discharge. MAR may be a promising and easily measurable marker for predicting short‐term SE outcomes.

## 6 Clinical Implications

SE is a life‐threatening neurological emergency with considerable mortality and morbidity. Our study indicated that MAR, a new inflammatory biomarker, could predict SE outcomes. SE patients with a high MAR at admission may face an elevated risk of unfavorable outcomes. Therefore, early monitoring of MAR may be vital to allow for timely interventions and improve SE outcomes.

## Author Contributions

F. J. and L. J. designed the study. P. L. L. performed the data acquisition. F. J. and L. J. L. carried out the statistical analysis. The manuscript was written by F. J. and reviewed by L. J. and Z. S. All authors read and approved the manuscript to be published.

## Funding

Our work was funded by the Youth Program of the Natural Science Foundation of Sichuan Province (No. 2025ZNSFSC1642) and the Strategic Cooperation Project of Luzhou Municipal People's Government‐Southwest Medical University (NO.2024LZXNYDJ052).

## Ethics Statement

The ethics committee of the affiliated hospital of Southwest Medical University approved the present study (Approval No.KY2024477), and this study was performed in agreement with the Helsinki Declaration principles.

## Consent

Written consent was acquired from the individual patient or his/her family members.

## Conflicts of Interest

The authors declare no conflicts of interest.

## Supporting information



Supplementary Table: brb371204‐sup‐0001‐TableS1.doc

## Data Availability

The data used to support the findings of this study are included within the article.
