# Supplementary material for: Monocyte‐to‐Albumin Ratio Predicts the Functional Outcome of Adults With Status Epilepticus: An Observational Study
Source: Brain Behav. 2026 Jan 15;16(1):e71204. doi: 10.1002/brb3.71204 (PMC12808917; doi:10.1002/brb3.71204)
Supplement: Supplementary file 1 — Supplementary Table: brb371204‐sup‐0001‐TableS1.doc [file BRB3-16-e71204-s001.doc]

**Supplementary Table 1 Clinical characteristics and poor functional outcomes of matched cohort.**

|  | **MAR** | | ***P*** |
| --- | --- | --- | --- |
|  | **< 13.78 (n=35) ≥ 13.78 (n=35)** | |  |
| Male (n, %) | 21 (60.0) | 23 (65.7) | 0.621 |
| Age, years, median (IQR) | 50.0 (29.0-63.0) | 59.0 (30.5-70.5) | 0.213 |
| SE etiology (n, %) 0.621 | | | |
| Cryptogenic | 12 (34.3) | 14 (40.0) |  |
| Symptomatic | 23 (65.7) | 21 (60.0) |  |
| Stroke etiology of SE (n, %) | 3 (8.6) | 4 (11.4) | > 0.999 |
| Infectious etiology of SE (n, %) | 7 (20.0) | 5 (14.3) | 0.526 |
| Fever at onset (n, %) | 12 (34.3) | 9 (25.7) | 0.434 |
| No history of seizures (n, %) | 14 (40.0) | 13 (37.1) | 0.806 |
| STESS at SE onset, median (IQR) | 2.0 (2.0-3.0) | 2.0 (2.0-4.0) | 0.109 |
| END-IT score, median (IQR) | 1.0 (1.0-2.0) | 1.0 (1.0-2.0) | 0.359 |
| Comorbidities (n, %) |  |  |  |
| Hypertension | 8 (22.9) | 14 (40.0) | 0.122 |
| Diabetes mellitus | 2 (5.7) | 4 (11.4) | 0.669 |
| Previous stroke | 2 (5.7) | 4 (11.4) | 0.669 |
| Poor outcomes at discharge (n, %) | 4 (11.4) | 23 (65.7) | < 0.001 |

IQR, interquartile range; SE, status epilepticus; STESS, status epilepticus severity score; END-IT, encephalitis-NCSE-diazepam resistance-image

abnormalities-tracheal intubation; MAR, monocyte-to-albumin ratio.
